# Effect of Fungal Solid-State Fermented Product in Broiler Chicken Nutrition on Quality and Safety of Produced Breast Meat

**DOI:** 10.1155/2018/2609548

**Published:** 2018-09-10

**Authors:** Slavomír Marcinčák, Tatiana Klempová, Martin Bartkovský, Dana Marcinčáková, Nevijo Zdolec, Peter Popelka, Ján Mačanga, Milan Čertík

**Affiliations:** ^1^Department of Food Hygiene and Technology, University of Veterinary Medicine and Pharmacy in Košice, Komenského 73, 041 81 Košice, Slovakia; ^2^Institute of Biotechnology, Faculty of Chemical and Food Technology, Slovak University of Technology, Radlinského 9, 812 37 Bratislava, Slovakia; ^3^Department of Pharmacology and Toxicology, University of Veterinary Medicine and Pharmacy in Košice, Komenského 73, 041 81 Košice, Slovakia; ^4^Department of Hygiene, Technology and Food Safety, Faculty of Veterinary Medicine, University of Zagreb, Heinzelova 55, 10 000 Zagreb, Croatia

## Abstract

The aim of this work was to analyse the effect of addition of 10% (w/w) fermented bioproduct into commercial broiler feed on fatty acid profile, lipid oxidative stability, and sensory properties of chicken breast meat. The fermented bioproduct was prepared by fermentation of cornmeal by filamentous fungi* Umbelopsis isabellina* CCF 2412 in solid-state fermentation (SSF) process and the final bioproduct was enriched with gamma-linolenic acid and beta-carotene. In the experiment, 80 pieces of 1-day-old chickens COBB 500 were used. Half of them (control group) were fed only with commercial feed. Chickens of the experimental group were fed with commercial feed, and, from the 11^th^ day of age until the time of slaughter (39^th^ day), 10% of commercial feed was replaced with fermented bioproduct. Application of fermented bioproduct into commercial feed mixture positively influenced profile of fatty acids in breast meat. The amount of gamma-linolenic, alpha-linolenic, and oleic acids in fat of breast muscles was increased and n-6/n-3 ratio was significantly decreased. Profile and content of PUFAs did not change after thermal treatment of meat. Oxidative stability of fat and sensory properties of the meat during the storage (4°C, 7 days) of meat were not affected by fermented bioproduct.

## 1. Introduction

Cereals are a staple food used in human and livestock nutrition. They are the most important energy sources and they provide various macronutrients (carbohydrates, proteins, dietary fibre, and lipids) and some micronutrients (vitamins, polyphenols, and minerals) for consumers [1]. However, cereals lack a variety of essential nutrients, especially polyunsaturated fatty acids (PUFAs) and carotenoid pigments [2, 3]. PUFAs are structural components of cell membranes and they regulate flexibility, fluidity, and permeability of membranes. Also, they play a key role in mammalian metabolism as a precursor of metabolites controlling critical biological function such as prostaglandins, leukotrienes, and thromboxanes [4]. Insufficient dietary consumption of PUFAs may lead to physiological imbalances of organisms, which may manifest in the form of diabetes, hypertension, inflammatory response problems, etc. [5]. Carotenoid pigments show many protective effects against malfunctions related to oxidative stress, such as cardiovascular and cancer diseases [6].

Solid state fermentation (SSF) is the oldest known fermentation technique. It imitates the natural environment of the microorganism. It involves a solid matrix that serves a dual function: as support and as a source of nutrients for microbes kept under conditions with a limited amount of free water [7]. During microbial growth, the solid matrix is enriched with various types of metabolites according to the employed microbe. Due to a limited amount of water present in the SSF process, the most promising class of microbes to be used are filamentous fungi [8, 9]. Moreover, the oleaginous lower filamentous fungi belonging to the* Zygomycetes* family (e.g.,* Thamnidium, Cunninghamella, Mortierella, Umbelopsis, *and* Mucor*) are very well known as producers of various PUFAs (e.g., gamma-linolenic acid and arachidonic acid) and carotenoid pigments in submerge as well as solid-state fermentation process [10–13]. Thus, SSF is one of the most qualified techniques for natural enrichment of solid material, such as cereals, with desired metabolites. One of the biggest advantages of material (bioproduct) obtained after SSF is that it can be used directly as a supplement for human or livestock nutrition without any further downstream processing, which improves the economy of the whole process.

Chicken meat is currently the most preferred thanks to its low cost, ease of culinary preparation, and its nutritional characteristics. In fact, broiler breast meat supplies high protein and low fat intakes [14]. This meat contains a higher proportion of unsaturated fatty acids in comparison to the meat of other slaughter animals. The fatty acid composition of poultry meat can be significantly influenced by feeding [14]. For example, one can add a suitable source of PUFAs into the feed [15].

The main challenge of our research is to determine the possibility of usage of fungal SSF bioproducts enriched with gamma-linolenic acid (GLA; C18:3, n-6), beta-carotene, and furthermore with coenzyme Q10, ergosterol, and amylase as supplements for broiler chicken feed. The purpose of this study was set to assess effect of feeding of 10% of prefermented bioproduct enriched with GLA and beta-carotene on quality and safety of produced breast meat.

## 2. Materials and Methods

### 2.1. Preparation of Fermented Feed

The strain* Umbelopsis isabellina* CCF2412 was obtained from Culture Collection of Fungi, Charles University, Prague, Czech Republic. This strain was described as a producer of both GLA and beta-carotene [11]. The culture was maintained on potato-dextrose agar (PDA; Carl Roth, Germany) at 4°C and reinoculated every 3 months. The spore suspension for inoculation of SSF was prepared from a 7-day-old culture grown on polished rice (28°C). The spores were washed with sterile distilled water with 0.05% (v/v) of Tween 80 and the spore suspension was diluted to a final concentration of 2.10^5^ spores/mL. The substrate for SSF, cornmeal, was obtained from company Amylum Slovakia (Boleráz, Slovak Republic). HDPE bags were filled with 100 g of cornmeal, moistened with distilled water (ratio 1:1) for 2 hours, and then autoclaved at 105°C for 1 hour. The cooled substrate was inoculated with 20 mL of spore suspension (prepared as described above). SSF was run for 7 days at 28°C. After fermentation, the obtained bioproduct was dried at 50°C until constant weight was achieved. The prepared dry bioproduct was packed and sent to University of Veterinary Medicine and Pharmacy in Košice (Slovak Republic) for bird feeding.

### 2.2. Birds, Housing, Diets, and Experimental Design

The animal protocol for this research was approved by the Ethical Committee for Animal care and Use of University of Veterinary Medicine and Pharmacy in Kosice. The experiment was carried out in accordance with the “European Directive on the protection of vertebrate animals used for experimental and other scientific purposes” [16] and with the consent of the State Veterinary and Food Administration of the Slovak Republic no. 309013-221 in the premises of Clinic for birds and exotic animals of The University of Veterinary Medicine and Pharmacy in Košice (Slovak Republic).

A total of 80 pieces of 1-day-old male broiler chickens of the meat type hybrid COBB 500 were obtained from Hydina Slovensko Broiler Company (Slovakia). The chicks were randomly divided into two groups of 40 birds (control and experimental group). Control and experimental group had four replicates. Each replicate contained 10 birds.

Chickens were reared on deep litter (wood shavings). Temperature and lightning regimes were in accordance with standards for the fattening of broiler chickens [17]. During the entire fattening period the broiler chickens had free access to water and feed. Feeding of chickens was carried out as follows: fattening period lasted 38 days and the conventional feed mixtures (DeHEUS, Czech Republic), which are used in industrial factory farms of broilers in Slovakia, were fed in both groups. Chickens of the control group were fed only with conventional feed mixtures used in the four phases. The main components of feed mixtures are wheat, corn, soybean meal, rapeseed cake, and sunflower meal. At the beginning of fattening, feed mixture “BR1” (starter diet; Crude protein 22.4%, Fat 6.2%, Fibre 3.2%, Ash 5.4%, Methionine 0.6%, Vit. A 12500 IU, Vit. D_3_ 4000 IU, and Vit. E 75 mg.kg^−1^, Ca 0.8%, P 0.58%) was administered during the first 10 days of fattening. From day 11 to day 21, birds consumed a growing diet 1 (BR2; Total protein 20.9%, Fat 6.2%, Fibre 3.4%, Ash 4.5%, Methionine 0.52%, Ca 0.6%, P 0.5%, Vit. A 10000 IU, Vit. D_3_ 4000 IU, Vit. E 25 mg.kg^−1^). From day 22 to day 28 birds were fed growing diet 2 (BR3; Total protein 20.3%, Fat 6.9%, Fibre 3.1%, Ash 4.2%, Methionine 0.5%, Ca 0.5%, P 0.45%, Vit. A 10000 IU, Vit. D_3_ 4000 IU, and Vit. E 25 mg.kg^−1^) and from 29^th^ to the 38^th^ day final diet (BR4; Total protein 19.3%, Fat 6.6%, Fibre 3.1%, Ash 3.9%, Methionine 0.48%, Ca 0.5%, P 0.41%, Vit. A 8000 IU, Vit. D_3_ 2000 IU, and Vit. E 20 mg.kg^−1^). Chickens of the experimental group were fed in the same way, but fermented bioproduct was administered from the 11^th^ day of fattening. Bioproduct was added into the feed mixture “BR2, BR3, and BR4” in the amount of 10% (10% of conventional feed mixture was replaced with bioproduct).

### 2.3. Sampling Procedures

On the 39^th^ day, the chickens were, after being stunned, bled and their carcasses were dressed. Subsequently, samples of breast muscle were collected. To determine the fatty acid profile, breast tissue samples (16 in each group) were packaged in polyethylene bags and stored in the freezer for analysis (maximum up to 1 month). For determination of the stability of the FA after heat treatment, half of the samples (8 pieces) were heated at 80°C in the core of the meat for 20 minutes. To detect antioxidant activity and oxidative changes of fats during storage of breast muscle samples in the refrigerator, samples (16 pieces per group) were packaged in polyethylene bags and stored in a refrigerator at 4°C for 7 days. For sensory analysis, samples of breast muscle (16 pieces per group) were packed into containers and evaluated 24 hours after slaughtering and after storage of sample for 7 days under chilled conditions (4°C).

### 2.4. Chemical Composition of Meat

Water content was determined by oven-drying at 105°C [18]. Kjeltec Auto, type 1030 analyser (Tecator Co., Hoganas, Sweden), was used to determine the crude protein content. Lipids were isolated in ground samples with petroleum ether in Soxhlet apparatus (LTHS 500, Brnenská Druteva v.d., Czech Republic) and were determined gravimetrically [18].

### 2.5. Fatty Acid Composition

Fatty acids were analyzed as their methyl esters (FAMEs) using gas chromatography. The methyl esters were prepared from lipid extract obtained from Soxhlet extraction. 2 mg of total lipid was diluted in hexane and 2-step esterification was performed using sodium methoxide in the first step and a solution of hydrochloric acid in methanol in second. The reaction was run for 1 hour, and the samples were consecutively centrifuged (5 min, 3000 x* g*) in order to separate organic phase. Organic phase containing methyl esters of fatty acids were transferred into vial and analyzed by GC-6890N (Agilent Technologies) [19]. FAMEs were evaluated by retention times of internal standard (Sigma, USA) and quantified by ChemStation B0103 (Agilent Technologies).

### 2.6. Determination of Lipid Oxidation and Antioxidant Activity of Meat

The extent of lipid oxidation was evaluated as thiobarbituric acid reactive substances (TBARS) [20]. A minced sample (1.5 g) was weighed in the test tube, 1 ml of 0.3% Ethylene Diamine Tetra Acetate was added and gently mixed with the sample, then 5 ml of 0.8% Butylhydroxytoluene in hexane was added, and the content of the test tube was mixed again. Shortly before homogenization, 8 ml of ice-cold 5% Trichloracetic acid (TCA) was added and homogenized for 30 seconds at 10.000 rpm (IKA S 18N-19G, Germany). After homogenization, the sample was left for 10 minutes and centrifuged for 5 minutes (3500 g, 4°C). Subsequently, the top hexane layer was removed, the sample was filtered (Whatman 4) and added to a 10 ml 5% TCA in a graduated flask. One ml of 0.8% thiobarbituric acid was added to 4 ml of sample in the test tube. Samples were incubated in a water bath for 30 minutes at 70°C. TBARS values were measured spectrophotometrically at 532 nm (Helios *γ*, v. 4.6 Thermo Spectronic, Cambridge, UK). TBARS values were determined within 24 hours after slaughter and after 7-day storage in refrigerator (4°C). TBARS results were quantified as malondialdehyde (MDA) equivalents and expressed as milligrams of malondialdehyde per kilogram of sample (mg.kg^−1^).

To determine the antioxidant activity of the breast muscles, a 3 g of sample was weighed into a 50 ml centrifuge test tube; 15 ml of trichloroacetic acid (TCA) was added and homogenized (3500 rpm) for 1 minute [21]. Subsequently, 10 ml of chloroform was added and the test tube shaken vigorously two-three times. The lipids and aqueous supernatant were separated by centrifugation (3500 rpm) for 5 minutes at 4°C. TCA supernatant was used to determine the antioxidant activity. Antioxidant activity was measured spectrophotometrically by the method of 2,2-diphenyl-1-picrylhydrazyl (DPPH) scavenging [22]. The DPPH solution in methanol (0.1 *μ*M, 0.0025 g/100 mL) was prepared fresh each day prior to analysis. 3.8 ml of the prepared solution was placed into a 1 cm thick cuvette (the extinction coefficient (A_DPPH_) was hence determined). Then, 200 *μ*l of the prepared meat extract was added and mixed for 10 minutes to react and absorbance was measured at 515 nm for up to 10 min to determine A_sample_.

The radical-scavenging capacity of meat extracts was calculated according to the following:

(1) $$\begin{array}{c} \%\;inhibition=\frac{A_{DPPH}-A_{sample}}{A_{DPPH}} \end{array}$$

### 2.7. Sensory Evaluation

Paired comparison test [23] was used to assess the sensory characteristics of the meat. Seven evaluators, who were trained in the field of descriptive analysis and terminology, were selected from the employees of the Institute of Hygiene and Meat Technology of UVLF in Košice. Samples of the meat were evaluated 24 hours after slaughter and storage in a refrigerator (4°C). The samples were heat-treated by the cooking method, achieving and maintaining an internal temperature of 70°C for 10 minutes. After cooking, the samples were cooled to 40°C, and they were administered to the panellists to assess the sensory properties. A 5-point rating system was used for each evaluated property: flavour, taste, juiciness, and tenderness (maximum 20 points).

### 2.8. Statistical Analysis

Statistical analysis was carried out using Graph Pad Prism 4.0 software [24]. Results for fatty acid profile of feed and breast meat are presented as the mean of four replicates determinations ± standard deviations (SD). Results for chemical composition, lipid oxidation, antioxidant activity, and sensory evaluation are presented as the mean of eight samples ± SD from each group. Individual results between groups were statistically compared with a one-way ANOVA test. A Tukey comparison test was used to compare statistical differences between the values of the monitored groups and* P* < 0.05 was considered a statistically significant difference.

## 3. Results and Discussion

### 3.1. Fatty Acid Profile of Feeds

The profile of fatty acids of fermented bioproduct (FB), commercial feed as well as the mixture of FB with commercial feed was in focus of our research. The results of analysis are in Table 1. The data shows that during SSF process the cornmeal was successfully enriched with both GLA and even with beta-carotene. The commercial feed mixture lacked these compounds. Both bioactive compounds were found in feed mixture that was provided to experimental group of birds. Thus, the intake of GLA and beta-carotene was secure during the whole experiment.

Further comparison of obtained data shows that employing of FB in commercial feed mixture not only enriched the mixture but modified the composition of other fatty acids. Commercial feed mixture contained a high percentage of medium-chain length saturated fatty acids, namely, lauric acid (C12:0) and myristic acid (C14:0). These fatty acids increased the growth rate and fattening rate of the birds; however, they have negative impact on bird metabolism since they cause elevation of cholesterol content in blood [25]. FB did not contain any lauric acid and only a minimal amount of myristic acid. Addition of FB into commercial feed mixture led to decreasing of content of these fatty acids (*P* < 0.05). Mixture of FB and commercial feed also exhibited lower content of alpha-linolenic acid (ALA; C18:3, n-3) (*P* < 0.05) and increased content of palmitoleic acid (*P *< 0.05) in comparison to conventional commercial feed.

It is known that composition of fatty acids of feed had impact on composition of fatty acid of produced meat [26]. Grain feed components contain little PUFAs, especially n-3 PUFAs, so there is an effort to enrich the feed with significant content of fatty acids. In order to increase n-3 PUFAs content in feed, linseed, rapeseed, soybean, and oils, or fish oil, are most commonly applied [26–28]. All of these components increase the PUFAs share, but there is often also a reduction in the feed stability, because of the PUFAs oxidation; thus storage period is shortened and the storage conditions of the feed are stricter. Since the GLA-oil is presented in FB, there is an increased potential that it could undergo the oxidation reaction. During these reactions, the various radical oxygen species (ROS) are created. ROS have a serious negative impact on living cells and mammals leading to diseases such as cancer. As mentioned above, carotenoid pigments have been described as effective antioxidant agents due to ability to scavenge the oxygen radicals [29]. Thus, the presence of beta-carotene in FB sustained its oxidation stability of PUFAs and protects the birds from ROS.

### 3.2. Chemical Composition of Meat

In the experiment, chemical analysis of breast muscle (after slaughter, dressing and heat treatment at 80°C for 20 minutes) was performed. The feed of the experimental group with the addition of 10% FB had an effect on the chemical composition of the chicken breast muscle. Compared with the control group, a lower water and fat content (*P* < 0.05) and a higher total protein content (*P *< 0.05) in the meat of the experimental group were recorded. Heat treatment affected the composition of the samples in both groups (Table 2). Compared to raw meats, both groups experienced a drop in water content and increased fat and total protein content. When comparing the chemical composition of the meat after the heat treatment, the lower fat content was found in experimental group compared to the control. The total protein content was higher in the experimental group, but statistical differences were not recorded between the groups (*P* > 0.05). It is concluded that feeding 10% FB has had an effect on reducing the fat content in chicken breast muscle. This is confirmed by the results obtained in the previous experiment [30], after feeding 5% fermented feed (prepared by fungal SSF using* Cunninghamella elegans*), a lower percentage of fat in the breast muscle was recorded while the total protein content was not affected with fermented feed. In contrast, other studies indicate that the fat supplements used in poultry feed (flax and fish oil) did not have a statistically significant effect on the fat content of the breast muscle [27, 31].

### 3.3. Fatty Acid Profile of Produced Breast Meat

The nutritional value of breast meat is also determined by the composition of fatty acids presented in meat. For better prediction of nutrition impact, the fatty acid composition has been determined in raw and cooked (boiled in water, 100°C, 20 min) breast meat. The results are shown in Table 3. The presented results confirm the claim that by varying the composition of FA in feed fats it is possible to influence the composition of FA of fats in produced meat [32]. A significant increase in GLA, ALA, and oleic acid was recorded in the fat of the chicken breast muscle of the experimental group after feeding 10% of the FB, and in addition the n-6/n-3 PUFAs ratio decreased. Feeding 10% FB in this experiment showed an increase in fatty acids corresponding to the composition of FA in feeds. The only exception is ALA, the proportion of which was higher in the experimental group compared to the control group, despite the fact that it was lower in the feed of the experimental group. From our point of view, there is a substantial increase in the GLA concentration, which was also the goal of our experiment. Similarly, it is possible to change the proportion of other PUFAs in the fat depending on the fat source used. The feeding of* Camelina sativa* oil as an ALA source resulted in an increase in ALA in chicken breast; on the contrary, an increase in linoleic acid was recorded after feeding with sunflower oil [28].

In the experiment, after feeding 10% FB, a decrease in the ratio of n-6/n-3 PUFAs in the fat of the breast muscle was recorded. This improved ratio is not a result of an increase in n-3 PUFAs, but mainly by a decrease in arachidonic acid (AA). A more significant reduction in the FB ratio was not predicted, as the organic product delivers mainly n-6 PUFAs to feed mixtures. The improved ratio in fat of the experimental group may also be a result of other changes that occurred during fermentation. The content of digestible starch dropped and the content of resistance starch increased [33]. The resistance starch is one of the main components of fibre that has been known as a positive influencer not only on lipid metabolism, but also on the whole physiology of organisms [34, 35]. It is complicated to compare the effect of FB on the composition of FA in fat of breast muscles with other results, since a similar type of fermented feed and a 10% concentration have not been used in chickens' nutrition yet. The fermentation substrate, but mainly the lower filamentous fungi used, has a significant effect on the production of significant metabolites (PUFAs, antioxidants, and enzymes). In our previous experiments we did not notice such a significant decrease in arachidonic acid, but the ALA fraction was higher than in this experiment [30, 36]. However, a different concentration (3 and 5%) was used in both experiments, and also different lower filamentous fungi (*Thamnidium elegans* or* Cunninghamella elegans*) were used for SSF and they produced only GLA.

Thermal treatment, cooking of the samples at 100°C for 20 minutes, varied the content of multiple fatty acids in both groups as compared to raw meat (Table 3). The most significant are the changes in AA and docosahexaenoic acid (DHA, C22:6, n-3). On the other hand, the content of other PUFAs, namely, GLA, ALA, and eicosapentaenoic acid (EPA), was not influenced by thermal treatment. This suggests that the final products of PUFAs biosynthesis (AA for n-6 and DHA for n-3) have lower stability than their precursors. This is the first time that such results have been obtained and other experiments (differential thermos-oxidant capacity measurement, detailed analysis of changes in membrane and storage lipids) are necessary to confirm such data.

### 3.4. Determination of Lipid Oxidation and Antioxidant Activity of Meat

The effect of the fermented bioproduct that was fed to the poultry on the antioxidant activity and the fat oxidation of meat (expressed as malondialdehyde content) during storage for 7 days in the refrigerator is shown in Table 4. The results show that the antioxidant activity of the meat was not affected by the feeding of 10% FB and the results of the ability to scavenge-free DPPH radical was comparable in the control and experimental groups on the first and also the 7^th^ day of storage of the samples in the refrigerator. Sample storage for 7 days in the refrigerator caused a decrease in antioxidant activity of the meat (*P* < 0.05) in both groups.

The results of fat oxidation, expressed as the amount of malondialdehyde, the major secondary product of PUFAs oxidation, were equal levels for both the control and the experimental group on the first day of storage of the samples in the refrigerator. After storage of the samples for 7 days in the refrigerator, the fat oxidation in both groups increased, but statistically significant differences (*P* > 0.05) were not found compared to control group. Oxidation changes in the experimental group were recorded at the same level as the control. Oxidation of the fat in breast muscle was not affected feeding 10% FB. The same oxidative stability of the meat of the experimental group, despite the higher unsaturated fatty acids content, especially GLA and ALA, is an important finding for this work. Regarding lipid oxidation stability of the meat, the results obtained in other studies indicate that as portions of MUFA and PUFAs in chicken meat increased, the susceptibility of meat to lipid oxidation also increased over the storage time [31]. However, oxidative changes of fats can be reduced by the addition of antioxidants in feed. Nkukwana [37] stated that the meat of chickens after feeding oil extracted from* Moringa oleifera* leaves had much better stability than the control meat attributed to the presence of natural antioxidants from the leaves. Likewise, in the fermented product, beta-carotene was produced by fermentation as an effective antioxidant, which had an effect on improving the oxidative stability of the meat. Similarly, supplementation of broiler diets with different levels of pomegranate byproducts, a rich source of natural antioxidants, significantly reduced the oxidation of lipids of breast meat [25].

### 3.5. Sensory Evaluation

The sensory evaluation of chicken breasts 24 hours after slaughter is shown in Table 5. In the sensory analysis of the meat samples, the following parameters were evaluated: flavour, taste, juiciness, and tenderness, with the maximum score in the monitored parameter being 5. The total maximum score for each sample was 20 points. The meat of the experimental group was evaluated on the 1st day of storage and was found to be better than the control meat (*P* > 0.05). After 7 days of storage, the meat of the experimental group was evaluated again and deemed better than that of the control. From the evaluated parameters, the juiciness and the flavour of meat were evaluated better compared to the control (*P* > 0.05). The results show that FB feeding had a positive effect on the sensory properties of the meat produced from the breast muscle. What is important, however, is that any negative impact of FB on the rated parameters was not recorded. This confirms the trend from our previous experiments after addition of 3 and 5% FB [30, 36], when we have similarly seen either a positive effect, or no differences were recorded in comparison with control meat. When using other sources of PUFAs in poultry diet, no negative impact on the sensory parameters of the meat was noted. The nonsignificant effect of 10 % flaxseed on sensory characteristics has also been reported [38]. However, in contrast, there is a study that reported a significant reduction in the aroma, flavour, taste, and overall acceptability of nugget meat from chickens fed with increasing levels of dietary flaxseed [39]. There is no literature on the sensory attributes of broiler meat being affected by feeding 10% FB available.

## 4. Conclusions

This work demonstrates the possibility of using the oil-producing lower filamentous fungi and SSF for the production of a fermented bioproduct (FB), which, by feeding to poultry, can significantly improve the quality and oxidative stability of the fat in produced meat. The FB prepared on the basis of cornmeal increased the GLA share and improved the ratio of n-6/n-3 PUFAs in raw meat. The stability of the fatty acids was maintained even after the heat treatment. It is also important to note that FB has improved the sensory properties of the produced meat. The oxidative stability of the meat during storage in the refrigerator was not affected by FB feeding. SSF feed production is an interesting form of production of significant fatty acids and beta-carotene, important ingredients that can be used to enrich the poultry diet and which subsequently increase the share of significant PUFAs and the oxidative stability of fats in the produced meat.

## Figures and Tables

**Table 1:** Profile of basic fatty acids and beta-carotene content in feeds.

		Grower diet 2		Finisher diet	
Fatty acids (%)	FB	Control	Experimental	Control	Experimental
C 12:0	S	10.65 ± 0.48	8.70 ± 0.60	10.95 ± 0.48	9.02 ± 0.16
C 14:0	0.52 ± 0.09	4.09 ± 0.25	3.44 ± 0.10	4.18 ± 0.69	3.30 ± 0.33
C 16:0	16.61 ± 0.71	12.86 ± 0.81	14.07 ± 0.51	14.36 ± 0.74	14.81 ± 0.34
C 16:1 n-7	1.49 ± 0.30	0.37 ± 0.01	0.72 ± 0.05	0.68 ± 0.03	0.86 ± 0.01
C 18:0	2.85 ± 0.43	3.28 ± 0.10	3.21 ± 0.04	4.49 ± 0.13	4.23 ± 0.16
C 18:1 n-9	40.50 ± 2.41	30.26 ± 0.26	33.04 ± 0.29	30.10 ± 0.38	32.43 ± 0.27
C 18:2 n-6	31.19 ± 4.28	30.80 ± 0.95	29.10 ± 0.26	28.09 ± 1.12	28.20 ± 0.68
C 18:3 n-6, GLA	3.49 ± 0.58	nd	1.14 ± 0.06	nd	0.79 ± 0.06
C 18:3 n-3, ALA	0.75 ± 0.12	3.05 ± 0.03	2.53 ± 0.18	2.77 ± 0.11	2.38 ± 0.19
ΣSFA	21.19 ± 1.16	31.63 ± 0.59	30.39 ± 0.08	34.56 ± 1.85	32.21 ± 0.89
ΣMUFA	43.94 ± 4.65	33.21 ± 0.28	36.05 ± 0.19	33.52 ± 0.48	35.86 ± 0.16
ΣPUFA	35.43 ± 3.82	33.85 ± 0.92	32.77 ± 0.28	30.86 ± 1.22	31.36 ± 0.69
Σn-6	34.68 ± 3.72	30.80 ± 0.95	30.25 ± 0.21	28.09 ± 1.12	28.98 ± 0.72
Σn-3	0.75 ± 0.12	3.05 ± 0.03	2.53 ± 0.18	2.77 ± 0.11	2.38 ± 0.19
Σn-6/Σn-3	46.71 ± 4.09	10.09 ± 0.39	12.04 ± 0.95	10.13 ± 0.12	12.28 ± 1.02
GLA, mg.g^−1^	3.034 ± 1.290	nd	0.626 ± 0.086	nd	0.432 ± 0.046
beta-carotene, *µ*g.g^−1^	3.12 ± 0.71	nd	0.55 ± 0.03	nd	0.52 ± 0.05

s: traces; nd: not detected; FB: fermented bioproduct; GLA: gamma-linolenic acid; ALA: alpha-linolenic acid; SFA: saturated fatty acids; MUFA: monounsaturated fatty acids; PUFA: polyunsaturated fatty acids.

**Table 2:** Chemical composition of breast muscles.

	Moisture (%)	Total lipids (%)	Crude protein (%)
Control	75.22 ± 0.15_ _^a^	3.50 ± 0.20_ _^ab^	22.02 ± 0.40_ _^b^
Experimental	73.95 ± 0.51_ _^b^	2.62 ± 0.31_ _^b^	23.00 ± 0.42_ _^a^
Control TT	73.77 ± 0.15_ _^b^	3.84 ± 0.34_ _^a^	22.87 ± 0.38_ _^a^
Experimental TT	73.37 ± 0.18_ _^b^	3.02 ± 0.16_ _^b^	23.24 ± 0.52_ _^a^

TT: thermal treated (80°C, 20 min); _ _^a, b^ : values with different superscripts in column are statistically different, *P* < 0.05.

**Table 3:** Profile of fatty acids in lipids of breast muscle.

Fatty acid (%)	Control	Experimental	Control TT	Experimental TT
C 16:0	21.62 ± 0.12	21.43 ± 0.39	22.24 ± 0.56	22.15 ± 0.01
C 16:1 n-7	0.50 ± 0.02	0.49 ± 0.08	0.55 ± 0.07	0.47 ± 0.06
C 18:0	9.43 ± 0.26_ _^a^	9.12 ± 0.66_ _^a^	7.51 ± 0.26_ _^b^	7.56 ± 0.06_ _^b^
C 18:1 n-9	30.31 ± 0.53_ _^c^	33.45 ± 0.59_ _^b^	35.19 ± 0.65_ _^a^	35.23 ± 0.16_ _^a^
C 18:1 n-7	3.88 ± 0.28	3.22 ± 0.13	3.28 ± 0.15	3.45 ± 0.06
C 18:2 n-6	16.34 ± 0.13_ _^a^	15.62 ± 0.27_ _^b^	15.47 ± 0.17_ _^b^	14.91 ± 0.06_ _^c^
C 18:3 n-6 (GLA)	0.119 ± 0.014_ _^b^	0.194 ± 0.003_ _^a^	0.137 ± 0.15_ _^b^	0.195 ± 0.007_ _^a^
C 18:3 n-3 (ALA)	0.819 ± 0.046_ _^b^	1.148 ± 0.023_ _^a^	1.070 ± 0.15_ _^a^	1.058 ± 0.028_ _^a^
C 20:2 n-6	0.487 ± 0.078	0.470 ± 0.060	0.438 ± 0.026	0.400 ± 0.014
C 20:3 n-3	0.081 ± 0.015	0.080 ± 0.020	0.086 ± 0.018	0.091 ± 0.021
C 20:3 n-6	1.20 ± 0.05_ _^a^	0.78 ± 0.05_ _^b^	0.67 ± 0.06_ _^c^	0.75 ± 0.01_ _^b^
C 20:4-n-6 (ARA)	5.75 ± 0.03_ _^a^	4.84 ± 0.25_ _^b^	2.31 ± 0.25_ _^d^	2.95 ± 0.03_ _^c^
C 20:5 n-3	0.373 ± 0.047_ _^a^	0.264 ± 0.014_ _^b^	0.349 ± 0.026_ _^a^	0.250 ± 0.23_ _^b^
C 22:5 n-3	0.193 ± 0.010_ _^a^	0.162 ± 0.025_ _^ab^	0.134 ± 0.026_ _^b^	0.167 ± 0.008_ _^b^
C 22:6 n-3	0.655 ± 0.024_ _^a^	0.465 ± 0.030_ _^b^	0.242 ± 0.020_ _^c^	0.268 ± 0.013_ _^c^
∑ SFA	34.41 ± 0.39	34.02 ± 0.07	34.62 ± 0.40	33.94 ± 0.77
∑ UFA	65.72 ± 0.43	66.02 ± 0.18	65.52 ± 0.41	66.06 ± 0.67
∑ PUFA n-3	2.12 ± 0.07_ _^a^	2.12 ± 0.05_ _^a^	1.88 ± 0.03_ _^b^	1.83 ± 0.07_ _^b^
∑ PUFA n-6	23.89 ± 0.19_ _^a^	21.90 ± 0.03_ _^b^	19.03 ± 0.52_ _^c^	19.20 ± 0.17_ _^c^
n-6/n-3	11.27 ± 0.28_ _^a^	10.33 ± 0.31_ _^b^	10.12 ± 0.11_ _^b^	10.48 ± 0.24_ _^b^

SFA: saturated fatty acid; UFA: unsaturated fatty acid; PUFA: polyunsaturated fatty acids; GLA: gamma-linolenic acid; ALA: alpha-linolenic acid; AA: arachidonic acid; TT: thermal treated (80°C, 20 min);  ^a,b,c  ^: values with different superscripts in column are statistically different, *P* < 0.05.

**Table 4:** Antioxidant activity and decomposition changes of fat in breast muscle stored for 7 days at 4°C.

	Day 1		Day 7	
	Control	Experimental	Control	Experimental
AA (%)	43.13 ± 1.57_ _^a^	43.16 ± 1.12_ _^a^	34.39 ± 1.20_ _^b^	33.48 ± 0.75_ _^b^
MDA (mg.kg^−1^)	0.101 ± 0.016_ _^b^	0.101 ± 0.008_ _^b^	0.129 ± 0.023_ _^ab^	0.154 ± 0.029_ _^a^

AA: antioxidant activity of meat; MDA: decomposition of fats expressed as a malondialdehyde content.

__^a, b^ : values with different superscripts in column are statistically different, *P* < 0.05.

**Table 5:** Sensory evaluation of breast muscle on days 1 and 7 of storage in refrigerator (points).

	Day 1		Day 7	
	K	FF	K	FF
Flavour	3.44 ± 0.53	3.56 ± 0.73	3.50 ± 0.67	3.89 ± 0.78
Taste	3.78 ± 0.44	3.78 ± 0.67	3.90 ± 0.75	3.78 ± 0.67
Juiciness	2.56 ± 0.56	3.34 ± 0.67	2.90 ± 0.42	3.78 ± 0.57
Tenderness	3.22 ± 0.97	3.33 ± 0.71	3.70 ± 0.72	3.44 ± 0.52
Sum	13.00 ± 1.50	14.00 ± 2.00	14.00 ± 1.30	14.89 ± 1.45

## Data Availability

The data used to support the findings of this study are included within the article.
